# miR-186 and 326 Predict the Prognosis of Pancreatic Ductal Adenocarcinoma and Affect the Proliferation and Migration of Cancer Cells

**DOI:** 10.1371/journal.pone.0118814

**Published:** 2015-03-05

**Authors:** Zheng-liang Zhang, Zheng-hai Bai, Xiao-bo Wang, Ling Bai, Fei Miao, Hong-hong Pei

**Affiliations:** Emergency Department, The Second Affiliated Hospital of Xi’an Jiaotong University, 710004, Xi’an, Shaan Xi, Peoples’ Republic of China; University of Nebraska Medical Center, UNITED STATES

## Abstract

MicroRNAs can function as key tumor suppressors or oncogenes and act as biomarkers for cancer diagnosis or prognosis. Although high-throughput assays have revealed many miRNA biomarkers for pancreatic ductal adenocarcinoma (PDAC), only a few have been validated in independent populations or investigated for functional significance in PDAC pathogenesis. In this study, we correlated the expression of 36 potentially prognostic miRNAs within PDAC tissue with clinico-pathological features and survival in 151 Chinese patients. We then analyzed the functional roles and target genes of two miRNAs in PDAC development. We found that high expression of miR-186 and miR-326 predict poor and improved survival, respectively. miR-186 was over-expressed in PDAC patients compared with controls, especially in patients with large tumors (>2 cm), lymph node metastasis, or short-term survival (< 24 months). In contrast, miR-326 was down-regulated in patients compared with controls and displayed relatively increased expression in the patients with long-term survival or without venous invasion. Functional experiments revealed that PDAC cell proliferation and migration was decreased following inhibition and enhanced following over-expression of miR-186. In contrast, it was enhanced following inhibition and decreased after over-expression of miR-326. A luciferase assay indicated that miR-186 can bind directly to the 3′-UTR of *NR5A2* to repress gene expression. These findings suggest that miR-186 over-expression contributes to the invasive potential of PDAC, likely via suppression of NR5A2, thereby leading to a poor prognosis; high miR-326 expression prolongs survival likely via the decreasing invasive potential of PDAC cells. These two miRNAs can be used as markers for clinical diagnosis and prognosis, and they represent therapeutic targets for PDAC.

## Introduction

Pancreatic ductal adenocarcinoma (PDAC) is the fourth leading cause of cancer-related deaths in the industrialized world and sixth in China [1,2]. Given a lack of distinct clinical manifestations and practical methods for early detection, most patients do not have the option of surgical resection when diagnosed [2]. Furthermore, approximately 70% of patients who receive surgery still undergo early recurrence within 6–12 months as a result of the highly aggressive properties of PDAC [3]. Currently, the overall 5-year survival of patients with PDAC is less than 5%, regardless of treatment [3]. Improving the PC mortality rate necessitates the discovery of new tools for the early detection, diagnosis, and monitoring of therapeutic efficacy.

Decades of studies revealed that persistent alterations in gene expression patterns due to epigenetic modifications, gene mutations and deletions are crucial for the development, progression, and maintenance of pancreatic tumors [4]. Although numerous changes in gene expression have been found in PDAC, it has been a challenge to identify the key genes that are responsible for the carcinogenesis and progression of pancreatic cancer. A strategy to screen out cancer-promoting genes or oncogenes is to correlate post-transcriptional biomarkers with clinico-pathological features and survival. A gene that significantly predicts cancer progression and prognosis may directly participate in carcinogenesis or interact with a gene that acts in the pathogenesis of cancer [5]. Moreover, identifying novel markers is helpful for developing new methods of early diagnosis and improving the dismal prognosis [6].

microRNAs (miRNAs) are a new family of small (typically 18–25 nucleotides), single-stranded non-coding RNAs. miRNAs bind specific target mRNAs in the 3′- untranslated region (UTR) with perfect or near-perfect complementarity, resulting in target mRNA degradation or translation inhibition, respectively. There are 2,042 mature human miRNAs currently identified that are predicted to regulate more than 30% of target human mRNAs [7]. Recently, increasing evidence demonstrates that miRNA deregulation contributes to carcinogenesis by promoting the expression of proto-oncogenes or by inhibiting the expression of tumor suppressor genes [8]. Such “onco-miRNAs” have also been demonstrated in PDAC: miR-196a promotes cancer progression by targeting NFKBIA, and miRNA-126 and 148a inhibit cancer cell growth by down-regulating CDC25B and ADAM9, respectively [9–12]. Moreover, many microRNAs were found to be aberrantly expressed in PDAC using expression profile analysis, although their functional roles in pancreatic carcinogenesis are still unclear [13–17]. Considering the deregulated gene expression networks in pancreas cancer [18], the post-transcriptional regulation for target gene in PDAC by miRNAs is worthy of further exploration.

The predictive significance of microRNA expression for clinico-pathological features and clinical outcomes of PDAC has also attracted extensive attention. Using high-throughput microarray chips, thousands of microRNAs have been evaluated in small samples of patients with PDAC, and a few were reported to be statistically significant biomarkers for survival [17,19,20]. Bloomston at el. profiled miRNA expression in 65 pairs of PDAC tissue and matched benign adjacent pancreatic tissue specimens [17]. They found that 21 miRNAs were up-regulated and four miRNAs were down-regulated in PDAC and that a subgroup of six miRNAs (miR-452, miR-105, miR-127, miR-518a-2, miR-187, and miR-30a-3p) was able to distinguish long-term survivors with node-positive disease from those who died within 24 months. Jamieson et al. associated miRNA expression profiles in 48 PDAC tissues with their clinicopathologic feature and prognosis and found that 20 miRNAs, including miR-21 and miR-34a, predicted overall survival [20]. It should be noted that the microRNA biomarkers uncovered by these studies are not consistent, which is partially attributed to the heterogeneity in characteristics of the patients as well as variable study designs and statistical analysis methods. Most biomarkers uncovered by these high-throughput studies have not been validated in other independent studies, and their functional role in PDAC development are also not clear [17,19,20]. These discrepancies highlight a need for the replication of the results in a large sample size to validate the predictive significances of those microRNAs. In the present study, we selected 36 miRNAs that were reported to be deregulated in PDAC or to significantly predict prognosis in previous studies and evaluated their predictive significance for survival in 151 Chinese patients with PDAC. Furthermore, we analyzed the functional role and target genes of two potential miRNA biomarkers in PDAC development.

## Materials and Methods

### 2.1 Patients and specimen collection

The present study enrolled 151 patients who underwent curative pancreatectomy at The Second Affiliated Hospital of Xi’an Jiaotong University during the period from 2005 to 2010. All patients were pathologically diagnosed with primary human pancreatic ductal adenocarcinoma (PDAC) according to the WHO classification. None of the patients had received preoperative chemotherapy. The demographic and clinico-pathological characteristics of the patients were obtained by reviewing the medical records and contacting the physicians in charge. The overall survival time was calculated from the time of diagnosis to death for pancreatic cancer deaths or the date of last contact or death from other causes for censored cases. A total of 151 formalin-fixed paraffin-embedded (FFPE) PDAC tissues from the resected tissue specimens of these patients were used for miRNA expression analysis. In addition, 15 cancer tissues and 14 adjacent normal pancreas tissues were obtained from the dissected surgery specimens of 15 patients with PDAC. All of the study procedures were approved by the Medical Ethics Committee of The Second Affiliated Hospital of Xi’an Jiaotong University (certificate No. MEC2012023). All of the study participants provided informed written consent.

### 2.2 miRNA selection

We reviewed the literature on the predictive significance of miRNAs for pancreatic cancer survival published from 2007 to 2012 in PubMed database. A total of 36 miRNAs were selected for validation in our patient cohort based on two criteria: 1) they were reported to be deregulated in PDAC or predict the survival of PDAC; 2) they were not validated in any independent patient cohort. The investigated miRNAs included miR-196a-2, miR-219, miR-452, miR-105, miR-127, miR-518a-2, miR-187, miR-30a-3p, miR-17–5p, miR-125b, miR-141, miR-154*, miR-181b, miR-186, miR-193, miR-221, miR-223, miR-224, miR-29c, miR-30a, miR-30c, miR-30d, miR-31, miR-33a, miR-34a, miR-378, miR-423, miR-455, let-7b*, miR-1207–3p, miR-1296, miR-1914*, miR-197, miR-326, miR-3610, miR-4290.

### 2.3 Cell Lines

The human PDAC cell lines, MiaPaca-2, BxPC3, Panc-1, and human pancreatic ductal epithelium (HPDE) cells were purchased from the Type Culture Collection of Chinese Academy of Sciences (Shanghai, China). The cells were grown in RPMI 1640 media (GIBCO, Life Technologies, Shanghai, China), supplemented with 10% FBS and 1% penicillin/streptomycin (Life Technologies, Shanghai, China). Cells were kept at 37°C with 5% CO_2_ in a humidified incubator and harvested with trypsin-EDTA in an exponentially growing phase.

### 2.4 RNA isolation and Quantitative RT-PCR

miRNAs were isolated from FFPE sections using a Qiagen miRNeasy FFPE Kit (Hilden, Germany) following the manufacturer’s protocol. Briefly, cancer tissues were macro-dissected from 4–6 slides of FFPE PDAC specimens after a review of the representative H&E-stained slides. The isolated tissues were deparaffinized with xylene and washed with ethanol and dried; they were then treated with proteinase K at 37°C overnight. The lysate was mixed with binding buffer RBC and transferred to a gDNA Eliminator spin column to remove genomic DNA. Then, a RNeasy MinElute column was used to bind total RNA from the remaining liquid. The purified RNA was eluted from that column by RNase-free water and was quantified by the absorbance at 260 nm with an ND-1000 spectrophotometer (NanoDrop, Wilmington, DE, USA). Total RNA was extracted from cultured cells and microdissected cells using a mirVana miRNA Isolation Kit (Ambion, Austin, TX, USA). The expression levels of each miRNA were examined with a TaqMan Quantitative Real-Time PCR (qRT-PCR) Assay using individual specific primers and probes according to the manufacturer’s instructions (Applied Biosystems, Foster City, CA, USA). For mRNA quantification, cDNA was synthesized from 2 μg of the large RNA fraction using the M-MLV reverse transcriptase, and real-time PCR analyses were conducted with SYBR *Premix Ex Taq* kit (TaKaRa Bio, Otsu, Japan). The primers for mRNA quantification were purchased from AuGCT, Inc. (Beijing, China), and the sequences are shown in S1 Table. The expression levels of each mature miRNA and mRNA were evaluated using the comparative threshold cycle (Ct) method and normalized to U6 snRNA (2^-ΔCt^). The fold change of each miRNA/mRNA was calculated from tumor tissues/cells versus normal tissues/cells and treated cells versus untreated cells. miRNA expression was determined to be high when the expression level was equal or above the mean of the cohort and low when it was below the mean of the cohort.

### 2.5 miRNA mimic and inhibitor transfection

Functional consequences of aberrant miR-186 and miR-326 expression were studied by transiently transfecting their mimics (miR-186 mimic and miR-326 mimic), inhibitors (Anti-miR-186 and Anti-miR-326, mirVana miRNA mimics/inhibitor, Life technologies, Shanghai, China), and corresponding negative controls (Anti-miR-NC and miR-NC for each miRNA, GenePharma, Shanghai, China) into MiaPaca-2, BxPC3, Panc-1 cells. Cells were seeded on a 24-well plate at 10,000 cells per well and transfected 24 hours later with an miRNA inhibitor or mimics at a final concentration of 10 nm using Lipofectamine 2000 (Invitrogen, Carlsbad, CA, USA) according to the manufacturer’s instructions. At 48 hours after transfection, cells were harvested for western blot or qRT-PCR analyses.

### 2.6 Cell Proliferation Assay

Cell proliferation was tested by colorimetric assay using a WST-1 reagent Kit (Roche Applied Science, Basel, Switzerland). A total of 5000 cells that had undergone 48 hours of transfection were placed into each well of a 24-well plate and incubated for an additional 48 hours. Then, 10 μl of WST-1 reagent was added and incubated for 3 hours. The absorbance at 450 nm was measured and compared with the reference value at 650 nm using a microplate reader and analyzed with SoftMax Pro 5 software. Each experimental group included at least 8 replicate wells, and all of the experiments were repeated at least three times independently. The relative cell proliferation was normalized with the corresponding control.

### 2.7 Transwell Cell Invasion Assay

BD Falcon 8.0-mm pore Transwell cell culture inserts (BD Biosciences, Franklin lakes, NJ, USA) were used to evaluate the invasiveness of PDAC cells transfected with different miRNAs or negative controls. The inserts were placed in a 24-well plate for 30 minutes before seeding cells. A total of 3× 10^4^ cells were placed in each well of the upper chamber and were incubated for 24 h at 37°C in 5% CO_2_. After incubation, migrated cells that remained on the bottom surface were fixed with a 4% paraformaldehyde (Sigma-Aldrich, Shanghai, China) solution for 10 minutes, washed with PBS and stained with 0.5% crystal violet (Sigma-Aldrich, Shanghai, China) for 10 minutes. Cell counting was performed through the use of a microplate reader. All of the experiments were performed independently in triplicate.

### 2.8 MiR-186 targets prediction

The putative miRNA targets were predicted by the TargetScan [21], miRanda [22], and PITA [23], RNAhybrid algorithms [24] using the default parameters included in the software for each. The putative target genes were further screened out by assessing the pancreatic expression database (http://www.pancreasexpression.org) [18]. We selected those genes that met two standards: 1) they were identified by all four algorithms; 2) they were revealed to be regulated along with PDAC development in previous studies.

### 2.9 Luciferase assay

To construct the green fluorescent protein (GFP) reporter plasmid, the 3′-UTR of fragment of the NR5A2 mRNA containing the predicted miR-186 binding site was amplified by PCR using the primers listed in S1 Table and inserted downstream of the GFP gene in pcDNA3.1/CT-GFP-TOPO vector (Promega, Madison, WI, USA). The resulting vector was named pGFP-NR5A2 3′-UTR. Site-directed mutagenesis of the miR-186 target site in the NR5A2 3′-UTR was carried out using a QuickChange mutagenesis kit (Stratagene, San Diego, CA, USA) with NR5A2 3′-UTR as a template and was named NR5A2 3′-UTR-Mut. In the mutated construct, the miR-186 target site 5′-ATTCTTT-3′ was substituted with a 5′-ACTGAAT-3′ fragment. NR5A2 3′-UTR-Mut was cloned into the pcDNA3.1/CT-GFP-TOPO plasmid to construct pGFP-NR5A2 3′-UTR-Mut. All of the insertions were confirmed by sequencing. MiaPaca-2, BxPC3, and Panc-1 cells were transfected with pGFP-NR5A2 3′-UTR or-Mut together with miR-186 mimics/Anti-miR-186 or the corresponding control vectors (miR-NC or Anti-miR-NC). All of the transfections were performed using Lipofectamine 2000 (Invitrogen, Carlsbad, CA, USA). Forty-eight hours after transfection, luciferase activities were measured by the Dual-Luciferase Reporter Assay System (Promega, Madison, WI, USA). Each experiment was repeated in triplicate.

### 2.10 Western blot analysis

Western blotting was performed to determine NR5A2 protein expression. The total protein was extracted from the PDAC tissues and normal tissues. Proteins were quantitated by the Bradford method according to the manufacturer’s protocol (Bio-Rad, Hercules, CA, USA). Samples (50 μg protein) were resolved on a 10% SDS-PAGE and transferred onto a nitrocellulose membrane. The membranes were probed with antibodies for NR5A2 and glyceraldehyde-3-phosphate dehydrogenase (GAPDH). All of the antibodies were purchased from Abcam company and were used at a dilution of 1:500 for anti-NR5A2 and 1:1000 for anti-GAPDH. The LabWorks image acquisition and analysis software (UVP, Upland, CA, USA) was used to quantify band intensities.

### 2.11 Statistical analysis

Survival curves were constructed with Kaplan—Meier analysis and compared using a log-rank test to assess the influence of individual miRNA and clinical covariate on outcome. We used a multivariate Cox proportional hazards mode to assess the overall survival risk via calculating the hazard ratio (HR) with 95% confidential intervals (CIs) with adjustment for competing risk factors in a backwards stepwise fashion. The variables with *P* < 0.1 on univariate analysis were included in multivariate analysis. A χ^2^ test was performed to make a comparison of the categorical variables. Student’s t-test, the Mann—Whitney U-test and analysis of variance were conducted to compare the continuous variables when appropriate. All of the *in vitro* experiments were performed in triplicate and were repeated at least two times, with data expression as the mean ± SD. All *P*-values are two sided and are considered statistically significant when less than 0.05. For multiple testing, false discovery rate (FDR) was calculated using the method described by Benjamini and Hochberg [25]. FDR estimates the proportion of the results that were declared positive that are actually false, and the results with FDR < 0.05 for multiple tests are considered statistically significant. Statistical analysis was conducted on SPSS 16.0 software.

## Results

### 3.1 Characteristics of the patients

**Fig 1:**
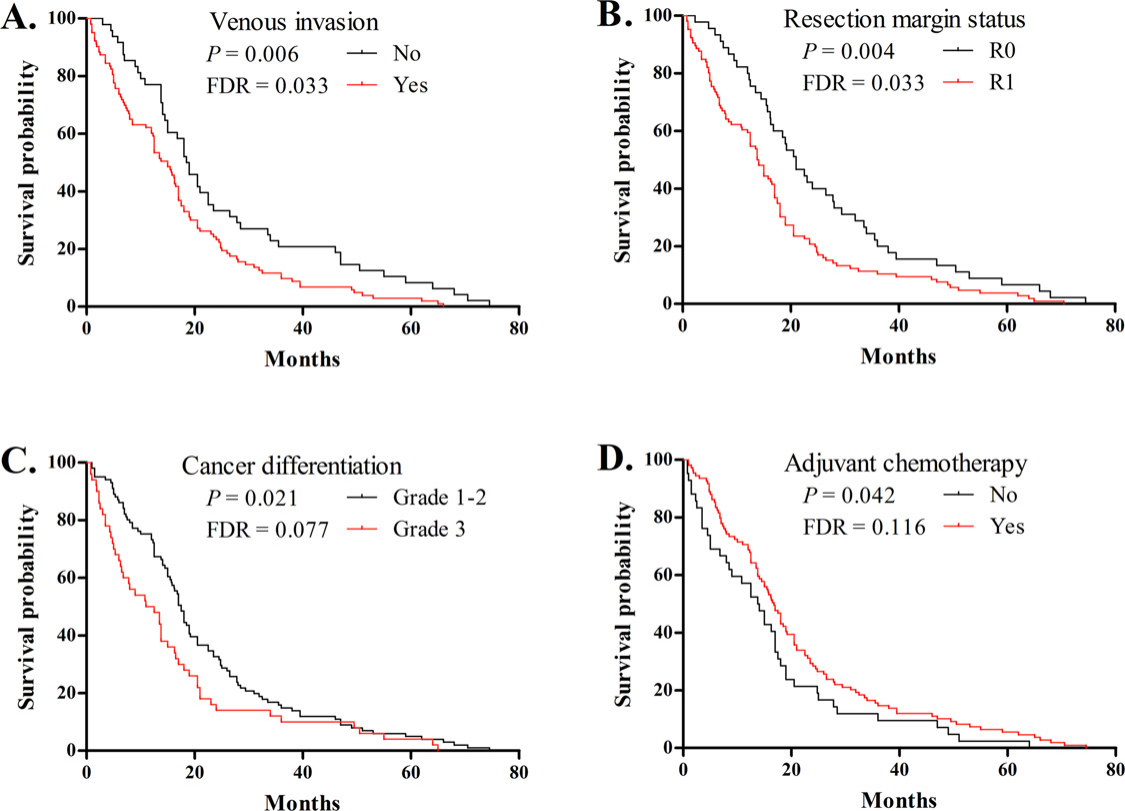
Kaplan-Meier curve for overall survival in patients with PDAC based on venous invasion (A), resection margin status (B), tumor differentiation (C), and adjuvant chemotherapy (D). Survival curves were compared by log-rank analysis for 11 demographic and clinico-pathological variables, and those with *P* < 0.05 are shown in this figure. FDR for each test result was calculated by the Benjamini and Hochberg method.

Table 1 summarizes the demographic and clinico-pathological characteristics of the 151 PDAC patients included in our study. Most patients had stage-T2/3 grade-1/2 tumors, with perineural and venous invasion, positive lymph nodes, and positive resection margins. The median overall survival was 16.2 months. Among these patients, 110 patients died within 24 months after resection (defined as short-term survivors), and 41 patients survived 24 months after resection (defined as long-term survivors). There were not significant differences in the demographic and clinico-pathological variables between short-term survivors and long-term survivors (FDR > 0.05, Table 1). Furthermore, univariate analysis for these 11 variables with a log-rank test revealed that poor overall survival was significantly associated with venous invasion (*P* = 0.006, FDR = 0.033, Fig. 1A) and resection margin involvement (*P* = 0.004, FDR = 0.033, Fig. 1B) but was nominally associated with tumor differentiation (*P* = 0.021, FDR = 0.077, Fig. 1C) and adjuvant chemotherapy (*P* = 0.042, FDR = 0.116, Fig. 1D). The clinico-pathologic factors with *P* value less than 0.10 were used for subsequent multivariate analysis.

**Table 1:** Demographic, clinico-pathological and treatment characteristics of PDAC resection patients stratified by the overall survival time.

Characteristic	All patients, n = 151	ST survivors^a^, n = 110	LT survivors^b^, n = 41	*P* value^c^	FDR
Demographic					
Gender (M/F)	90/61	67/43	23/18	0.592	0.977
Age of diagnosis, Mean±SD	57.8±9.75	57.9±9.85	57.3±9.57	0.734	0.977
(≤65/> 65)	108/43	78/32	30/11	0.784	0.977
Smoking history (no/yes)	52/99	38/72	14/27	0.963	0.977
Alcohol history (no/yes)	35/116	30/80	5/36	0.051	0.153
Pathologic					
Tumor grade (G1 or G2/G3)	101/50	68/42	33/8	0.030	0.153
Tumor size (≤2 cm/ >2 cm)	26/125	19/91	7/34	0.977	0.977
Lymph node metastasis (no/yes)	50/101	36/74	14/27	0.869	0.977
Perineural invasion (no/yes)	51/100	32/78	19/22	0.046	0.153
Venous invasion (no/yes)	48/103	32/78	16/25	0.244	0.586
Operative, treatment and outcome					
Resection margin status (R0/R1)	45/106	26/84	19/22	0.007	0.084
Adjuvant chemotherapy (no/yes)	42/109	33/77	9/32	0.326	0.652
Survival, month, (median)	16.2	12.5	36	—	—

^a^ short-term survivors have an overall survival time < 24 months.

^b^ long-term survivors have an overall survival time > 24 months.

^c^ *P* value was calculated by the 2×2 χ^2^ test (categorical variables) and Mann-Whitney U-test (age of diagnosis) and was corrected by the Benjamini—Hochberg FDR test.

—represents that it is not informative to compare. ST: short-term; LT: long-term.

### 3.2 miRNA expression in PDAC samples and their clinico-pathological and prognostic significance

We measured the expression levels of 36 miRNAs in the 151 FFPE tissues of PDAC by qRT-PCR and correlated their expression levels with clinico-pathological features. We found 1 miRNA differentially expressed based on tumor size, 2 for lymph node metastasis, and 3 for venous invasion after controlling for FDR < 0.05 (Table 2). Cancer tissues with a size that was greater than 2 cm expressed increased miR-186 than those that were less than 2 cm (*P* < 0.0001, FDR = 0.004). Lymph node metastasis significantly correlated with elevated miR-186 (*P* = 0.002, FDR = 0.030) and miR-224 (*P* = 0.0001, FDR = 0.002). Tumor progression stage had a significant association with increased miR-186 expression (stage I~II vs III~IV; *P* ≤ 0.0001, FDR = 0.004). Venous invasion was accompanied by increased miR-224 (*P* = 0.001, FDR = 0.012) and decreased miR-326 (*P* < 0.0001, FDR = 0.001) and miR-452 (*P* < 0.0001, FDR = 0.001).

**Table 2:** Differentially expressed microRNAs based on clinico-pathological characteristics.

Clinico-pathological characteristics	miRNA symbol^a^	Expression level comparison^b^			
		The group of patient		statistical outcome	
Tumor size		≤2 cm (n = 26)	>2 cm (n = 125)	*P* value	FDR
	miR-186	1.37±0.71	4.35±4.46	**< 0.0001**	**0.004**
	miR-196a	6.75±11.76	66.49±123.47	0.035	0.348
Lymph node metastasis		no (n = 50)	yes (n = 101)		
	miR-186	2.05±1.59	4.72±4.81	**0.002**	**0.030**
	miR-221	23.83±25.47	104.06±206.24	0.012	0.151
	miR-224	31.05±23.42	56.01±43.77	**0.0001**	**0.002**
Venous invasion		no (n = 48)	yes (n = 103)		
	miR-125b	27.39±20.04	18.65±11.20	0.008	0.074
	miR-224	33.30±19.29	54.46±45.09	**0.001**	**0.012**
	miR-326	40.52±57.76	14.40±31.79	**< 0.0001**	**0.001**
	miR-452	55.32±86.57	22.10±65.65	**< 0.0001**	**0.001**
Tumor stage		I~II(n = 54)	III~IV(n = 97)		
	miR-186	2.06±1.95	4.80±4.78	**< 0.0001**	**0.004**
	miR-224	33.68±24.62	55.37±44.46	0.0002	0.348

^a^ The table only includes the miRNAs that were expressed differently between the corresponding two groups with significant p-values < 0.05.

^b^ miRNA levels were expressed as the mean±SD. *P* value was calculated by the Mann—Whitney U-test and was corrected by the Benjamini-Hochberg FDR test. The outcomes with FDR less than 0.05 were in bold.

**Fig 2:**
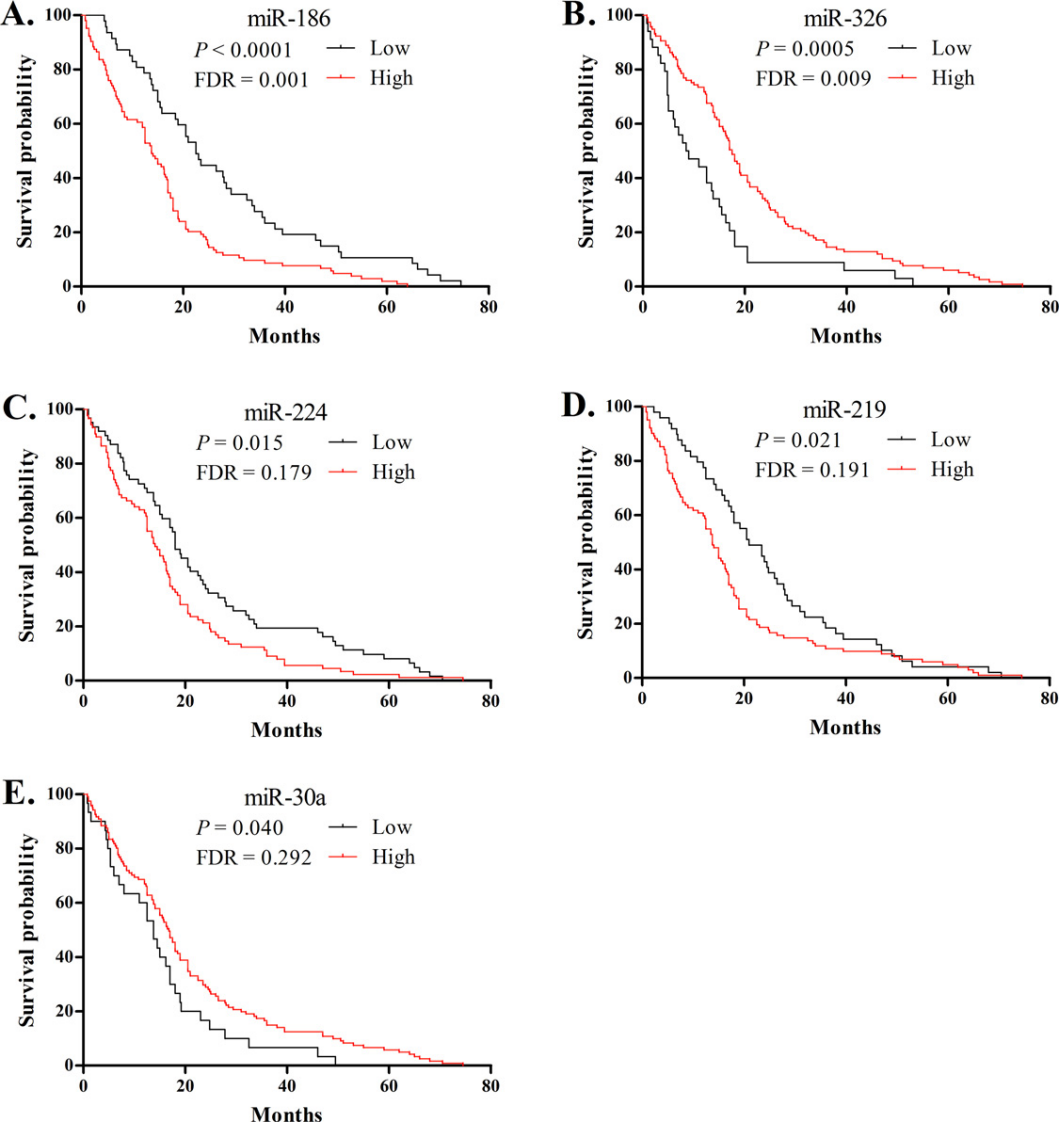
Kaplan-Meier curve for overall survival in patients with PDAC based on the binomial variable of high or low expression relative to the mean expression of miR-186 (A), miR-326 (B), miR-224 (C), miR-219 (D), miR-30a (E) using RT-PCR. Survival curves were compared by log-rank analysis for 36 miRNAs, and the ones with *P* < 0.05 are shown in this figure. FDR for each test result was calculated by the Benjamini and Hochberg method.

We subsequently evaluated the prognostic value of these 36 miRNA expression levels in PDAC using two methods described by Bloomston et al. [17]. First, we assessed the miRNAs that had an absolute expression level that could discriminate between short- and long-term survivors. We found that miR-186, miR-221, and miR-224 were differentially over-expressed in the patients with shorter survival, while miR-125b and miR-326 were over-expressed in the patients with longer survival with *P* values of 3.3×10^-6^–0.005 and FDR 0.0001–0.036 (Table 3). Next, we conducted a log-rank analysis to compare Kaplan-Meier survival curves of two patient groups that were defined as high or low expression relative to the mean expression of each miRNA on the qRT-PCR. Five miRNAs displayed nominal difference in survival curves regarding high vs. low expression (*P* < 0.05, Fig. 2), but only miR186 and miR-326 remained statistically significant after correcting for FDR. High expression of miR-186 significantly predicted a poorer prognosis (median survival of 13.8 months for high expression vs. 22.5 months for low expression, *P* < 0.0001, FDR = 0.001). High expression of miR-326 correlated with a better prognosis (median survival of 17.5 months for high expression vs. 8.8 months for low expression, *P* = 0.0005, FDR = 0.009). Finally, seven miRNAs with *P* < 0.1 in the previous two tests of survival were included in a multivariate model along with potential prognostic clinico-pathologic factors. The Cox proportional hazards regression analysis confirmed that high expression of miR-186 (HR = 1.655, 95%CI: 1.137–2.410, *P* = 0.009), miR-224 (HR = 1.445, 95%CI: 1.033–2.021, *P* = 0.032) and miR-221 (HR = 1.432, 95%CI: 1.007–2.036, *P* = 0.046) remained independent predictors of poor outcome, whereas high expression of miR-326 (HR = 0.476, 95%CI: 0.317–0.715, *P* < 0.001) and adjuvant chemotherapy (HR = 0.649, 95%CI: 0.443–0.950, *P* = 0.026) independently predicted better survival (Table 4).

**Table 3:** Differentially expressed microRNAs based on overall survival status.

MiRNA symbol^a^	Overall survival status		Statistical outcome	
	Short-term survivors	Long-term survivors	*P* value	FDR
miR-31	10.25±16.49^b^	5.57±8.56	0.034	0.209
miR-125b	18.45±11.02	29.43±20.81	**0.0002**	**0.004**
miR-186	4.55±4.69	1.91±1.30	**0.005**	**0.036**
miR-221	77.68±105.20	77.18±192.80	**0.005**	**0.036**
miR-224	53.79±44.48	31.48±14.86	**0.001**	**0.015**
miR-326	16.47±41.27	39.45±45.12	**<0.0001**	**<0.001**

^a^ The table only includes the miRNAs that were expressed differently between the corresponding two groups with significant p-values < 0.05.

^b^ miRNA levels were expressed as the mean±SD. *P* value was calculated by the Mann—Whitney U-test and was corrected by the Benjamini-Hochberg FDR test. The outcomes with FDR less than 0.05 are in bold.

**Table 4:** miRNAs and clinico-pathological factors associated with the overall survival of PDAC in multivariate analysis.

Multivariate analysis				
Covariates		Hazard ratio (95%CI)	df^a^	*P* value
Adjuvant chemotherapy	yes	0.649, 0.443~0.950	1	0.026
	no	1 (ref.)		
mir-186	high	1.655, 1.137~2.410	1	0.009
	low	1 (ref.)		
mir-224	high	1.445, 1.033~2.021	1	0.032
	low	1 (ref.)		
mir-326	high	0.476, 0.317~0.715	1	<0.001
	low	1 (ref.)		
mir-221	high	1.432, 1.007~2.036	1	0.046
	low	1 (ref.)		

### 3.3 Functional consequences of miR-186 and miR-326 regulation in human PDAC cells

**Fig 3:**
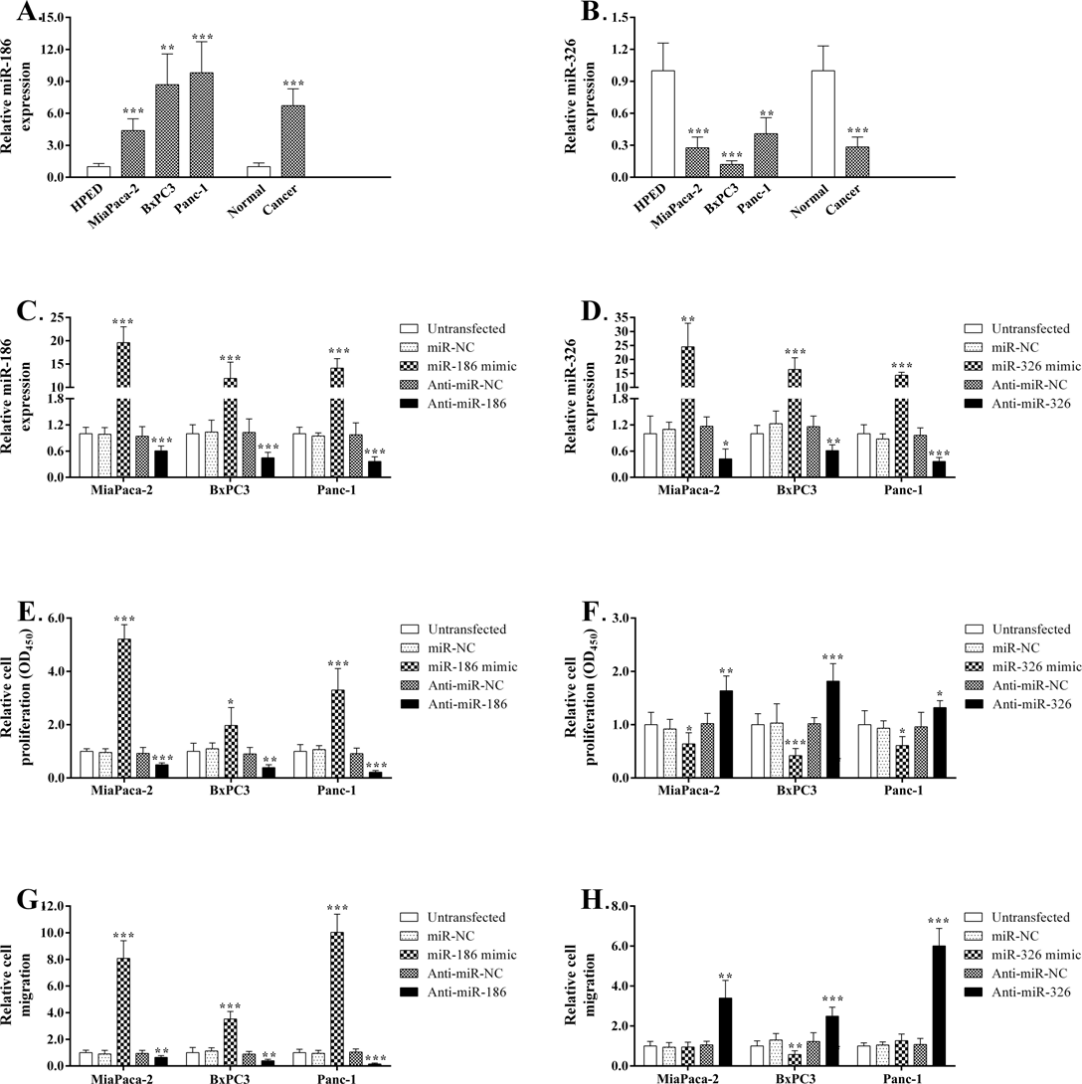
Functional analyses of miR-186 or miR-326 regulation in PDAC cell lines. Analysis of miR-186 (A) and miR-326 (B) expression in three PDAC cell lines, HPDE cells, PDAC tissues and adjacent normal pancreatic tissues. (C) Analysis of miR-186 expression in three PDAC cell lines after the transfection of the miR-186 mimic, Anti-miR-186, and respective negative controls. (D) Analysis of miR-326 expression in three PDAC cell lines after the transfection of the miR-326 mimic, Anti-miR-326, and respective negative controls. Cell proliferation was assessed in the PDAC cell lines transfected with miR-186 mimics, Anti-miR-186 or the corresponding negative control (E) and in the PDAC cell lines transfected with miR-326 mimics, Anti-miR-326 or the corresponding negative control (F) using the WST-1 assay. Relative cell growth was normalized to its respective control-treated cells. The graphs show relative cell migration after either inhibition or over-expression experiments for miR-186 (G) and miR-326 (H), as evaluated by the Transwell migration assay. Relative cell migration was normalized to its respective control-treated cells. Data presented are the mean of three independent experiments. Error bars represent standard deviations from the mean. *, *P* < 0.05, **, *P* < 0.01 and **, *P* < 0.001.

First, we measured the miR-186 and miR-326 levels in three PDAC cell lines, HPDE cells, PDAC tissues and adjacent normal tissues by qRT-PCR. Compared with normal pancreatic tissues or HPDE cells, miR-186 increased the expression level more than 4.4-fold both in PDAC tissue samples and cell lines. Conversely, miR-326 expression decreased more than 0.5-fold in both PDAC tissues and cell lines (Fig. 3A and B). Moreover, 48 hours after transfection in three PDAC cell lines, miR-186 and miR-326 were reduced by approximately 0.3–0.6-fold following the transfection of their inhibitors and were increased 5–30-fold after transfection with their mimics (Fig. 3C and D). Next we evaluated the effects of the down-regulation of these two miRNAs or their over-expression on cell proliferation, colony-formation, and migration in these three PDAC cell lines. We observed that inhibition of miR-186 expression in all three cell lines led to a significant decrease in cell growth (~50–79%; *P* < 0.01), while over-expression of miR-186 in these cells resulted in a significant increase in cell proliferation (~96–420%; *P* < 0.05) compared with the respective negative controls (Fig. 3E). In contrast to the miR-186 results, miR-326 inhibition caused a significant elevation in cell growth (~19–35%; *P* < 0.001), while its up-regulation by transfection brought about significant decrease of cell proliferation (approximately 21–32%; *P* < 0.01) compared with the respective negative controls (Fig. 3F). Taken together, both gain- and loss-of-function experiments consistently supported the contributing effects of miR-186 and the suppressive effects of miR-326 on PDAC cell proliferation. Transwell assays were performed to determine the effects of miR-186 or miR-326 regulation on cell migration. Our data indicated that the migration of all three PDAC cell lines was significantly enhanced via miR-186 over-expression that was induced by its mimic (approximate 250–900%; *P* < 0.001) and was inhibited by its suppression (approximate 35–62%; *P* < 0.01; Fig. 3G). In contrast, miR-326 over-expression resulted in a significant decrease in the migration of BxPC3 cells (approximate 43%, *P* < 0.01), and its suppression caused a significant elevation of migration in all three PDAC cell lines (approximate 150–500%, *P* < 0.001; Fig. 3H).

### 3.4 Validation of NR5A2 as a miR-186 Target Gene

**Fig 4:**
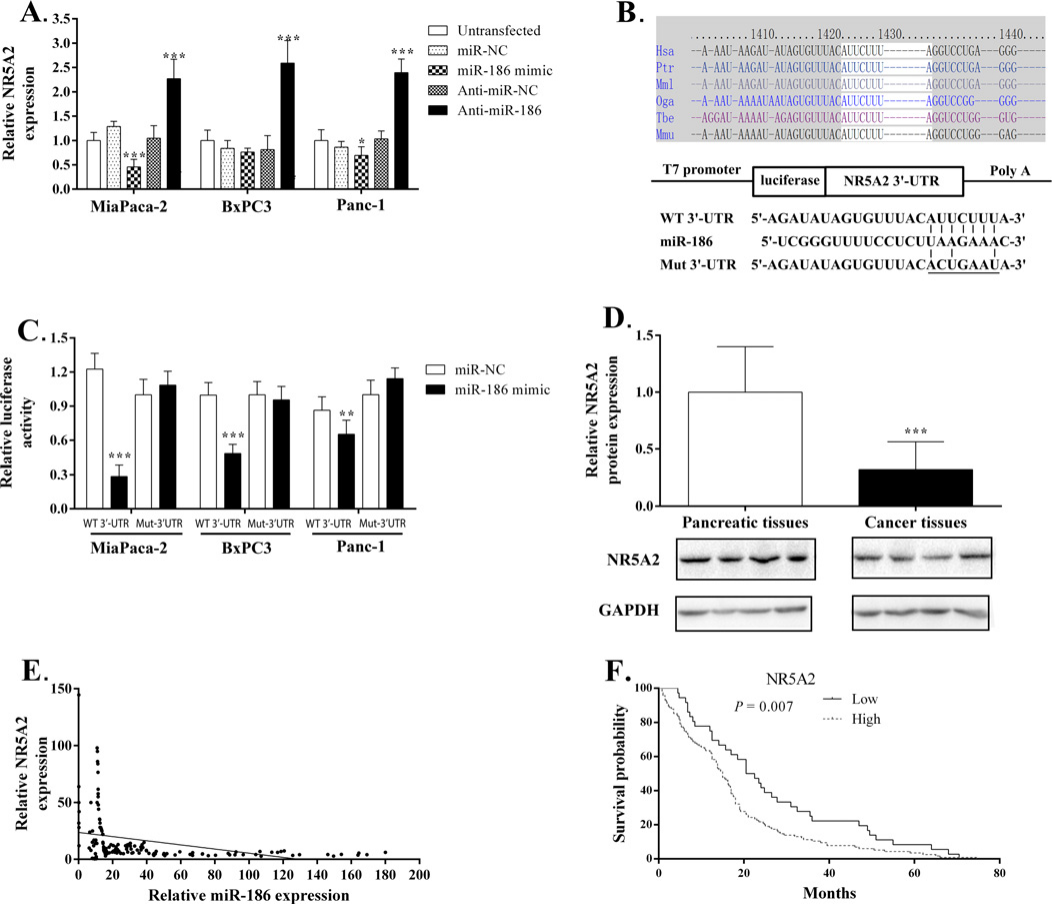
MiR-186 directly targets the NR5A2 gene. (A) qRT-PCR analyses of NR5A2 expression levels following the treatment of MiaPaca-2, BxPC3, Panc-1 cells with miR-186 inhibitors or mimics. (B) Human NR5A2 3′-UTR fragment containing a wild-type (WT) or mutant (Mut) miR-186–binding sequence was cloned downstream of the luciferase reporter gene. (C) The intensity of EGFP fluorescence was decreased in cancer cells transfected with both miR-186 mimics and NR5A2 WT 3′-UTR, but was unaltered in those transfected with both miR-186 mimics and the 3′-UTR mutant vector. (D) Relatively lower expression of NR5A2 was found in cancer samples compared with their normal counterparts using western blot assay. (E) There is an inverse correlation between the expression level of miR-186 and NR5A2. The expression relationship was evaluated by Pearson’s correlation analysis. *P* < 0.05 was considered statistically significant. *, *P* < 0.05, **, *P* < 0.01 and **, *P* < 0.001. Hsa, Homo sapiens; Ptr, Pan troglodytes; Mml Macaca mulatta; Oga, Otolemur garnettii; Mmu, mouse.

Three target genes for miR-186 (NR5A2, FAM150B, CTNND2) and six target genes for miR-326 (PLXNA2, CUGBP2, RUNX3, HSD11B1, PKIA, SULF1) were chosen by a combined search in bioinformatics databases of putative miRNA targets and the pancreatic expression database. Previous evidence suggested that these genes may be regulated during PDAC development. Multiple algorithms also predicted they may be targets for these two miRNAs. To determine whether they can be regulated by the two miRNAs, we first performed a qRT-PCR assay to observe the expression alteration of these genes in PDAC cells by each miRNA gain- and loss-of-function. Among these genes, only the NR5A2 gene displayed corresponding alterations in expression according to miR-186 over-expression and suppression. MiR-186 over-expression in MiaPaca-2, BxPC3, Panc-1 cells resulted in a significant decrease in NR5A2 expression (approximate 24–58%, *P* < 0.01). Inhibition of endogenous miR-186 expression in these cells significantly increased NR5A2 mRNA level (approximate 124–142%, *P* < 0.001; Fig. 4A).

Next, we used an EGFP reporter assay to validate the target site in the NR5A2 mRNA 3′-UTR. We constructed plasmids that contained EGFP and either a miR-186-binding site (pEGFP-NR5A2 3′-UTR) or a mutant site (pEGFP-NR5A2 3′-UTR-Mut) (Fig. 4B). We then observed whether miR-186 modulation by its mimics or Anti-miR-186 could regulate the EGFP reporter expression. When NR5A2 3′-UTR was transfected, the intensity of EGFP fluorescence was significantly reduced by miR-186 mimics in MiaPaca-2, BxPC3, Panc-1 cells. More importantly, the regulatory effects of the miR-186 mimics on EGFP fluorescence disappeared when 3′-UTR-Mut was co-transfected instead of 3′-UTR in cancer cells (Fig. 4C). Together, these results demonstrate that miR-186 binds directly to the 3′-UTR of NR5A2 to repress gene expression.

To further validate the *in vivo* expression relationship between miR-186 and NR5A2, we first evaluated NR5A2 expression in 14 normal pancreatic tissues and 15 PDAC tissues by western blot. We found significantly lower expression of NR5A2 in human PDAC samples compared with their normal counterparts (*P* = 0.006; Fig. 4D). Next, we correlated the NR5A2 mRNA level with miR-186 expression and survival. We found the expression patterns of NR5A2 were inversely correlated with the miR-186 expression (Corr = -0.358, *P* < 0.001; Fig. 4E), and its over-expression predicted an improved prognosis (Fig. 4F).

## Discussion

In the current study, we validated the predictive value of 36 miRNAs for PDAC clinic-pathological characteristics and prognosis in Chinese patients. We found that several miRNAs exhibited significant associations with tumor size (miR-186), lymph node metastasis (miR-186 and 224), venous invasion (miR-224, 326, and 452), and survival time (miR-125b, 186, 221, 224, and 326). miR-186 and 326 inspired further research as they consistently predicted multiple clinic-pathological features and survival with the most significant levels among identified miRNAs. Because little is known about the roles of these two miRNAs in carcinogenesis of PDAC, we subsequently investigated their function in PDAC cell proliferation and migration and sought to identify their specific target mRNAs.

With respect to miRNA-186, previous evidence has indicated that it is significantly up-regulated in pancreatic cancer tissue and several PDAC cell lines compared with normal control subjects [13] and that its over-expression can predict poor prognosis in patients with PDAC [20]. Consistently, we found that miR-186 was over-expressed in PDAC tissues and three cell lines and that its up-regulation was associated with larger tumor size, lymph node metastasis and shorter survival. These findings suggest that it is a potential oncogenic miRNA for PDAC. We further validated the oncogenic role of miR-186 in PDAC using an *in vitro* model and demonstrated that the gain- and loss-of-function of miR-186 exerted opposite effects on the growth and migration potential of PDAC cells. Up-regulation of miR-186 promoted the growth and migration of PDAC cells *in vitro*. This correlates with enhanced invasion and metastatic potential, which may indicate why miR-186 over-expression in cancer tissue correlated with a poor survival.

The oncogenic effects of miR-186 on PDAC development are in contrast to its effects on other tumors. In lung cancer, miR-186 down-regulation correlates with poor survival [26]. Forced over-expression of miR-186 in lung adenocarcinoma cells inhibits proliferation and invasion by targeting and inhibiting the cyclin D1, cyclin-dependent kinase 2 (CDK2), and CDK6 genes [26] and pituitary tumor transforming gene [27]. In prostate cancer, miR-186 often exhibits diminished expression, and its down-regulation correlates with the elevated expression of several prostate cancer-associated genes, such as alpha-methylacyl-CoA racemase and prostate-specific membrane antigen gene [28]. In colorectal cancer cells, miR-186 cooperates with other miRNAs to promote cellular senescence through the p53–p21^Cip1/WAF1^ pathway [29]. The difference in the carcinogenic effects of miR-186 among these cancers highlights that a specific pathogenesis underlies various cancers. The distinctive function of miR-186 in these cancers may be attributed to distinctive target genes in the development of these cancers. One miRNA is able to target multiple mRNAs to orchestrate the gene regulation of various biologic processes. Increasing evidence indicates that the functional targets of a miRNA can change depending on the specific pathophysiological context [30]. Identifying the target genes of miR-186 in various tumors will be a key method for understanding its oncogenic function. Based on bioinformatics analysis and reporter assays, we demonstrated that miR-186 can directly bind to the 3′-UTR of NR5A2 to repress gene expression.

In PDAC, miR-186 has an oncogenic role and represses NR5A2 expression, suggesting that NR5A2 may play a suppressive role in PDAC carcinogenesis. Recent evidence suggests that NR5A2 could contribute to PDAC through a role in the recovery from pancreatitis-induced damage [31–33]. NR5A2 participates in biliary acid metabolism and is a major regulator of the pancreatic exocrine program [32]. Moreover, single nucleotide polymorphisms in the vicinity of *NR5A2* are associated with a risk for PDAC [34]. Using *Nr5a2* knockout mice, Flandez et al. provided the first functional validation of *NR5A2* genetic variation as risk factor for pancreatic cancer. They found that a full *Nr5a2* dose is required to restore pancreatic homeostasis upon damage and to suppress the *KRas* ^*G12V*^-driven mouse pancreatic intraepithelial neoplasia progression, indicating that *Nr5a2* is a novel pancreatic tumor suppressor [31]. Our survival analysis revealed that high NR5A2 expression in cancer tissues predicted an improved prognosis, which also supported its protective roles in PDAC development. Considering the protective effect of NR5A2 on PDAC carcinogenesis and the direct suppressive effects of miR-186a on NR5A2 expression, we propose that NR5A2 may be a mediator for the oncogenic effects of miR-186 on PDAC. The interaction between miR-186 and NR5A2 in PDAC development needs to be further studied in the future.

With respect to miR-326, its high expression in cancer tissue predicted decreased odds to develop venous invasion as well as a better prognosis. Expression analysis revealed that miR-326 was down-regulated in PDAC tissues and cell lines. Previous studies indicated that miR-326 expression is decreased in advanced breast cancer tissues and in gliomas, suggesting that miR-326 may have tumor-suppressive effects on multiple tumors [35,36]. Benjamin et al. recently demonstrated that miR-326 has tumor-suppressive properties in glioblastoma cells by inducing apoptosis and reducing metabolic activity [37]. In line with their findings, we found that a miR-326 inhibitor or mimic consistently up or down-regulated cell proliferation and migration, respectively. Taken together, these data suggest a protective role for miR-326 in PDAC carcinogenesis. We failed to identify a target gene of miR-326 in PDAC, which underscores the importance of future analyses of its key downstream targets (such as proliferation and migration related gene).

In conclusion, we have shown in detail that miR-186 or miR-326 levels are importantly altered in PDAC tissues and PDAC cell lines. Moreover, miR-186 directly regulates NR5A2 and contributes to cellular proliferation and migration. The discovery of these miRNAs not only helps us to better understand the pathogenesis of PDAC but also provides new specific markers for PDAC prognosis, and treatment. It is important to note that our findings are mainly based on *in vitro* experiments, the biological roles of miR-186 and 326 in PDAC carcinogenesis and progression have not been revealed completely. Further studies need to confirm their regulatory effects on PDCA using *in vivo* xenograft models and investigate their roles in pancreatic normal cell line cycling status and proliferation.

## Supporting Information

S1 Table Primers used in this study. (DOC)
