# Supplementary material for: miR-186 and 326 Predict the Prognosis of Pancreatic Ductal Adenocarcinoma and Affect the Proliferation and Migration of Cancer Cells
Source: PLoS One. 2015 Mar 5;10(3):e0118814. doi: 10.1371/journal.pone.0118814 (PMC4351009; doi:10.1371/journal.pone.0118814)
Supplement: S1 Table — (DOC) [file pone.0118814.s001.doc]

**S1_Table**

Primers used in this study

| **Name** | **Sequence (5****' - 3')** |
| --- | --- |
| NR5A2 |  |
| Forward: | 5'-CTCATCCGAGCCAATGGACTT-3' |
| Reverse: | 5'-CAAGGCAGCATGGTTCAGA-3' |
| FAM150B |  |
| Forward: | 5'-CCCTCCGTTCCTGTCACTTG-3' |
| Reverse: | 5'-CTCACCCGCCCATCTCCT-3' |
| CTNND2 |  |
| Forward: | 5'- TTCATCACAGGTGCTGCGTAA-3' |
| Reverse: | 5'-CCATCACACTCTCTCATCCTTCTG-3' |
| CUGBP2 |  |
| Forward: | 5'- TGCTTCAACCCCCAACTCC-3' |
| Reverse: | 5'- GTCCTTGCAGAGTCCCGAGA-3' |
| HSD11B1 |  |
| Forward: | 5'- AGGAAAGCTCATGGGAGGACTAG-3' |
| Reverse: | 5'- ATGGTGAATATCATCATGAAAAAGATTC-3' |
| SULF1 |  |
| Forward: | 5'-CCACCTTCATCAATGCCTTT-3' |
| Reverse: | 5'-GGGAAGAGCAGTTCTCGTTG-3' |
| NR5A2 3'-UTR |  |
| Forward: | 5'- ATTAAGGAAATTACCGCTTTTG-3’ |
| Reverse: | 5'- CCGTAGCGTACAATTATTATGA-3’ |
| GAPDH |  |
| Forward: | 5'-TGC ACC ACC AAC TGC TTA GC-3’ |
| Reverse: | 5'-GGC ATG GAC TGT GGT CAT GAG-3’ |
